# Mechanistic Study of Schisandrin B in Alleviating Anxiety‐Like Behaviors in Rats via Enhancement of Hippocampal Synaptic Plasticity and Restoration of the Intestinal Barrier

**DOI:** 10.1002/fsn3.72315

**Published:** 2026-09-08

**Authors:** Jia‐Yu Tian, Rui‐Ze Wang, Hong‐Kun Wang, Xiao‐Nan Ye, Ruo‐Xuan Li, Zi‐Hao Wang, Li‐Li Huang, Yan‐Yan Wang

**Affiliations:** ^1^ Department of Pharmacology of Traditional Chinese Medicine Heilongjiang University of Chinese Medicine Harbin P. R. China

**Keywords:** AMPAR, anxiety, HPA axis, intestinal barrier, NMDAR, Schisandrin B, synaptic plasticity

## Abstract

This study focused on examining the anxiolytic effects of Schisandrin B (SchB) in rats exhibiting anxiety‐like behaviors and investigating the mechanisms involved. Behavioral assessments were conducted using the elevated plus‐maze test and the open field test. The results showed that Schisandrin B at a dose of 10 mg/kg significantly alleviated anxiety‐like behaviors in rats, with an efficacy comparable to that of the positive control drug diazepam. Mechanistic studies indicate that, at the central level, Schisandrin B effectively maintains the homeostasis of the glutamatergic system in the hippocampus and the HPA axis. Specifically, Schisandrin B downregulates the stress‐induced overexpression of ionotropic glutamate receptor subunits at both the mRNA and protein levels, including NMDAR subtypes (GluN1A, GluN2A, and GluN2B) and AMPAR subtypes (GluA1 and GluA2), thereby restoring the glutamate/γ‐aminobutyric acid (Glu/GABA) ratio to physiological levels. In addition, Schisandrin B suppresses the hyperactivation of the HPA axis by reducing the secretion of corticotropin‐releasing hormone (CRH), adrenocorticotropic hormone (ACTH), and corticosterone (CORT). Meanwhile, it ameliorates neuronal and synaptic ultrastructural damage in the hippocampus and upregulates the expression of synaptic plasticity–related proteins, SYP and PSD‐95, thereby improving synaptic transmission and function. At the peripheral intestinal level, Schisandrin B alleviated colon shortening in anxiety‐model rats and reduced fecal pH, thereby improving intestinal environmental homeostasis. Schisandrin B also promoted the upregulation of tight junction proteins (ZO‐1, Occludin, and Claudin‐1) in the colonic tissues, contributing to the restoration of colonic mucosal integrity, attenuation of inflammatory infiltration, and reinforcement of intestinal barrier function. Collectively, Schisandrin B exerts systemic anxiolytic effects by centrally regulating hippocampal glutamatergic neurotransmission, HPA axis activity, and synaptic plasticity, while simultaneously repairing peripheral intestinal barrier dysfunction. These results indicate the potential of Schisandrin B as a therapeutic candidate for anxiety disorders.

## Graphical abstract

Schisandrin B ameliorates stress‐provoked anxiety‐like behaviors in rats via restoring intestinal barrier tight‐junction integrity, balancing hippocampal glutamate‐GABA neurotransmission, and promoting hippocampal synaptic plasticity.

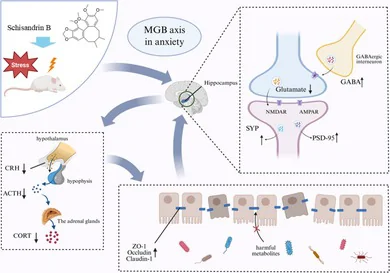

## 1 Introduction

Anxiety disorders, as highly prevalent mental health conditions worldwide, have become a major public health problem that seriously threatens both physical and mental well‐being. Their prevalence has shown a steady upward trend in recent years. Data from the World Health Organization indicate that anxiety disorders, due to their high prevalence and frequent comorbidity with various chronic diseases, have become the ninth leading cause of disability globally, exerting profound negative impacts on both affected individuals and society as a whole (Penninx et al. 2021). Anxiety disorders impose a substantial burden on patients' quality of life and socioeconomic status. However, commonly used clinical anxiolytic drugs are associated with limitations such as excessive sedation, gastrointestinal adverse effects, and high recurrence rates. In contrast, traditional Chinese medicines and natural bioactive compounds, characterized by multi‐target therapeutic actions and fewer side effects, have been identified as promising options for creating new treatments for anxiety disorders (Thibaut 2017).

The hippocampus is closely involved in the pathogenesis of anxiety disorders. Although the dorsal and ventral axes of the hippocampus have distinct functions, studies have shown that both participate in the regulation of anxiety, with the ventral hippocampus (vHPC) playing a particularly prominent role in anxiety modulation (Mikulovic et al. 2018; Shi et al. 2023; Wang et al. 2020). A study involving intra‐vHPC administration of a N‐methyl‐D‐aspartate receptor (NMDAR) agonist and antagonist found that NMDAR activation and inhibition produced anxiogenic and anxiolytic effects, respectively, thereby demonstrating the role of the hippocampus and the glutamatergic system in the regulation of anxiety (Rezvanfard et al. 2009). Neurons form neural circuits through synaptic connections, the strength of which can be dynamically regulated by neuronal activity—enhanced activity strengthens synaptic connections, whereas reduced activity weakens them. This regulatory capacity endows the nervous system with adaptability. Based on plasticity‐related mechanisms that modulate synaptic strength, synapses can undergo transient to long‐term morphological and functional changes, a key property known as synaptic plasticity (Wu et al. 2022). A study in which rats with chronic stress–induced anxiety were housed in an enriched environment showed that hippocampal synaptic plasticity was positively affected, thereby improving anxiety‐like behaviors (Bhagya et al. 2017). In the central nervous system, the dynamic equilibrium between glutamate (Glu) and γ‐aminobutyric acid (GABA) is essential for maintaining excitatory–inhibitory homeostasis, and disruption of this balance, together with dysfunction of associated receptors, is widely recognized as a critical pathological mechanism underlying anxiety disorders (Ren et al. 2024). Glutamate receptors (NMDARs and AMPARs), as core molecules mediating excitatory synaptic transmission, play critical roles in the regulation of synaptic plasticity, the development of anxiety, and emotional modulation. Their excessive activation can induce excitotoxicity and synaptic dysfunction, which are important contributors to the emergence of anxiety‐like behaviors (Rezvanfard et al. 2009). Therefore, the hippocampus plays a crucial role in the regulation of anxiety.

Meanwhile, the intestinal barrier, as an important defensive barrier of the body, when its integrity is compromised, can lead to increased intestinal permeability and leakage of inflammatory mediators. These changes indirectly affect central nervous system function through immune‐inflammatory responses, thereby exacerbating anxiety states. Accumulating evidence has demonstrated that dysfunction of the intestinal barrier is closely associated with the onset and progression of anxiety disorders (Ait‐Belgnaoui et al. 2012; Vancamelbeke and Vermeire 2017). Schisandrin B, as a core active component of *Schisandra chinensis*, has also been demonstrated to possess neuroprotective effects on the central nervous system and anxiolytic properties (Hu et al. 2012; Ko and Lam 2002). Nevertheless, the precise anxiolytic mechanisms of these two, particularly their synergistic effects and specific molecular targets involved in modulating glutamatergic pathways, enhancing synaptic plasticity, and protecting intestinal barrier function, remain to be systematically elucidated.

*S. chinensis* (Turcz. [Baill.]) is an important medicinal plant, and its fruit monograph has been included in the pharmacopeias of China, Japan, Korea, the United States, Russia, and the International Pharmacopeia (Sowndhararajan et al. 2018). Schisandrin B (SchB) is the most abundant dibenzocyclooctadiene lignan found in *S. chinensis*. Modern studies have demonstrated that SchB possesses a variety of pharmacological activities. One study investigated the effects of SchB on tert‐butyl hydroperoxide–induced neurotoxicity in mice and suggested that SchB counteracts oxidative stress in the brain through its antioxidant properties (Ko and Lam 2002). On the other hand, only highly lipophilic small‐molecule drugs are able to passively traverse the blood–brain barrier (BBB) through transcellular pathways (Gooyit et al. 2012), SchB, owing to its lipophilic nature and low molecular weight, can readily cross the BBB (Hu et al. 2012; Wang et al. 2018). A study on the effects of wine‐processed *S. chinensis* lignans (including SchB) on anxiety‐like behaviors showed that SchB exerts anxiolytic‐like effects by protecting hippocampal function while modulating the gut microbiota system (Song et al. 2021).

Based on the above research background, this study uses an anxiety rat model as the research object and focuses on three core targets—glutamatergic pathways, synaptic plasticity, and the intestinal barrier—to systematically investigate the anxiolytic effects of Schisandrin B and its mechanisms in improving intestinal barrier function. Behavioral tests were conducted to verify its anxiolytic efficacy. Molecular biology, electrophysiological, and morphological techniques were employed to explore its regulatory effects on the balance of Glu/GABA levels in the hippocampus, NMDARs, and AMPARs, along with synaptic structural and functional plasticity. In parallel, changes in intestinal barrier function were assessed, with particular attention to intestinal morphology. This research aims to explore the key molecular and physiological mechanisms by which Schisandrin B produces its anti‐anxiety effects and to elucidate the interconnected regulatory network involving the central glutamatergic pathway, synaptic plasticity, and intestinal barrier. This offers scientific validation and fresh theoretical insights for using traditional Chinese medicine and natural compounds in managing anxiety disorders.

## 2 Materials and Methods

### 2.1 Network Pharmacology Analysis of Schisandrin B

#### 2.1.1 Prediction of Potential Targets of Schisandrin B

The chemical structure and potential targets of Schisandrin B were retrieved using “Schisandrin B” as the keyword from the Traditional Chinese Medicine Systems Pharmacology Database (TCMSP, https://tcmsp‐e.com/tcmsp.php), SwissTargetPrediction database (http://www.swisstargetprediction.ch/), and PubChem database (https://pubchem.ncbi.nlm.nih.gov/).

#### 2.1.2 Screening of Disease‐Related Targets

Anxiety‐related targets were collected from the GeneCards database (https://www.genecards.org/) using “anxiety” as the keyword.

#### 2.1.3 Identification of Common Targets and Network Construction

The overlapping targets between Schisandrin B–related targets and anxiety‐related targets were identified using the Venny 2.1 online tool. These common targets were considered potential core targets through which Schisandrin B may exert its therapeutic effects.

#### 2.1.4 Protein–Protein Interaction (PPI) Network Analysis

The common targets were imported into the STRING database to construct a protein–protein interaction (PPI) network, with the species restricted to *Homo sapiens* and the minimum interaction confidence score set to 0.4. The PPI network data were downloaded and imported into Cytoscape software. Network topological parameters were analyzed using the NetworkAnalyzer plugin, and core targets were identified based on higher degree values.

#### 2.1.5 GO Functional Enrichment and KEGG Pathway Analysis

Gene Ontology (GO) functional enrichment analysis, including biological process (BP), cellular component (CC), and molecular function (MF), as well as Kyoto Encyclopedia of Genes and Genomes (KEGG) pathway enrichment analysis, was performed for the common targets using the DAVID database.

### 2.2 Molecular Docking Analysis of Schisandrin B

The three‐dimensional structures of key target proteins were obtained from the Protein Data Bank (PDB, https://www.rcsb.org/). Molecular docking analysis was conducted using the CB‐Dock2 online platform (https://cadd.labshare.cn/cb‐dock2/php/blinddock.php), including ligand preparation, protein preprocessing, and docking procedures. The binding affinities between Schisandrin B and the key targets were recorded, and the optimal compound–target binding modes with the highest affinities were visualized.

### 2.3 Animals

Specific‐pathogen‐free (SPF) male Sprague–Dawley rats (180–220 g; Animal License No. SCXK 2019–0004) were obtained from the GLP Laboratory Animal Center of Heilongjiang University of Chinese Medicine. Animals were maintained under standardized laboratory conditions, with the ambient temperature controlled at 23°C ± 1°C and a 12 h light/12 h dark cycle. Before the start of the experiments, all rats underwent a 7‐day acclimatization period. All procedures involving animals were conducted in accordance with the guidelines approved by the Animal Ethics Committee of Heilongjiang University of Chinese Medicine (Approval No. 2021013102).

### 2.4 Modeling and Drug Treatments

**FIGURE 1:**
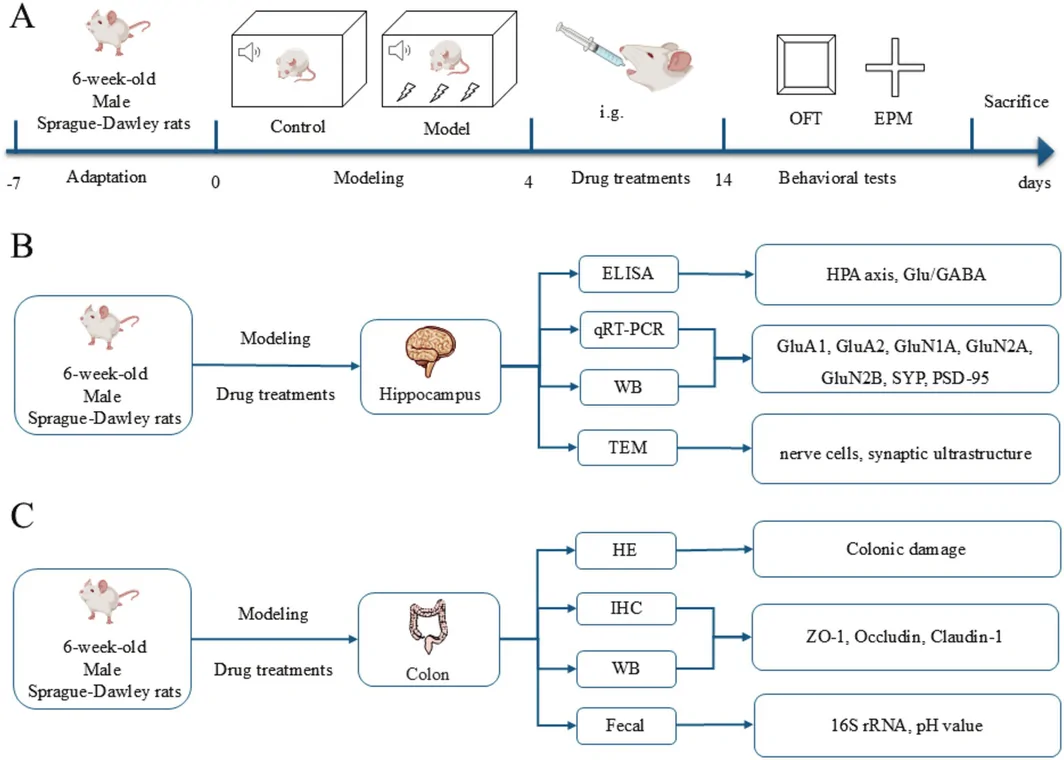
Behavioral assessments after pharmacological intervention, along with biomarker evaluations in the hippocampus and colon tissues, were conducted in rats exhibiting anxiety induced by conditioned fear. (A) Chronological protocol for rodent model establishment, pharmacological intervention, and behavioral phenotyping. (B) Biomarkers and analytical methods in rat hippocampal tissue. (C) Biomarkers and analytical approaches in rat colon tissue.

In the present study, a rat model of state anxiety was established over a 4‐day period (Yan et al. 2025). During the model induction period, animals were allowed to acclimate to the experimental setup for 3–5 min each day in order to reduce stress caused by handling and testing procedures. Male Sprague–Dawley rats were randomly divided into six groups (*n* = 8 per group): control, model, diazepam positive control (DZP, 5.2 mg/kg), Schisandrin B low‐dose (SchB‐L, 5 mg/kg), Schisandrin B medium‐dose (SchB‐M, 10 mg/kg), and Schisandrin B high‐dose (SchB‐H, 20 mg/kg). All treatments were administered by intragastric gavage once daily for 10 consecutive days. Except for the control group, all rats underwent foot‐shock conditioning. Briefly, after a 3‐min adaptation period in the conditioning chamber, animals were presented with an auditory cue (75 dB, 100 Hz) lasting 5 s. A 0.5 mA foot shock (1 s duration) was delivered simultaneously at the fifth second of the tone. This pairing procedure was repeated at 1‐min intervals over a 15‐min session. After completion of the conditioning protocol, rats remained in the chamber for an additional 3‐min habituation period. The control group received the same auditory stimulation but without foot shock. Following each session, the chamber was cleaned with 75% ethanol to remove residual odor cues. Behavioral assessments were conducted 24 h after the final drug administration. Schisandrin B (purity ≥ 95%, Batch No. DW0009) was supplied by Chengdu Letianmei Pharmaceutical Technology Co. Ltd., while diazepam tablets (Batch No. 20201112) were purchased by Shandong Xinyi Pharmaceutical Co. Ltd. The detailed experimental design is depicted in Figure 1.

### 2.5 Behavioral Tests

#### 2.5.1 Open Field Test (OFT)

After the final treatment, the open field test (OFT) was conducted. Rats were allowed to adapt to the testing environment for 1 h beforehand. Each animal was then carefully positioned in the center of a square open‐field arena. Their behaviors were tracked and analyzed automatically for 5 min using an intelligent video tracking system (Chen et al. 2023).

#### 2.5.2 Elevated Plus‐Maze Test (EPM)

The elevated plus‐maze test (EPM) was conducted immediately after the open field test. A video tracking system automatically recorded the number of entries into open arms and the time spent in open arms during the 5‐min test session. The percentage of time spent in open arms was calculated using the formula: (time in open arms/total movement time) × 100%. All experiments were performed in a sound‐attenuated behavioral testing room with controlled illumination conditions (Knight et al. 2021).

### 2.6 Sample Preparation

Based on the outcomes of behavioral experiments, we selected a dose of 10 mg/kg Schisandrin B for subsequent mechanism studies. After the behavioral tests, following a 12 h fasting period (with free access to water), experimental rats were anesthetized by intraperitoneal injection of 3% sodium pentobarbital (30 mg/kg). Under aseptic conditions, blood was collected from the abdominal aorta followed by rapid decapitation. The rats were immediately placed on a 0°C–4°C ice‐cooled operating platform for brain dissection. Bilateral hippocampal tissues were quickly isolated via microdissection, flash‐frozen in liquid nitrogen (< 30 s), and stored at −80°C for subsequent ELISA analysis. At the same time, freshly excreted fecal materials were aseptically obtained from the colonic lumen approximately 5 cm distal to the ileocecal junction using sterilized surgical tools.

### 2.7 Transmission Electron Microscope (TEM)

The hippocampal tissue was precisely dissected from rats using meningeal instruments according to standard stereotaxic techniques and immediately immersed in 2.5% glutaraldehyde solution for primary fixation. After tissue hardening, approximately 1 mm^3^ tissue blocks were carefully trimmed and subjected to systematic processing including post‐fixation, gradient dehydration, resin embedding, and ultrathin sectioning. The sections were then double stained with 3% lead citrate and 0.1% uranyl acetate (10 min each), followed by ultrastructural observation under a Nikon electron microscope (Japan) at three different magnifications: 11,000× for overall neuronal distribution in the hippocampal region, 21,000× for organelle ultrastructure, and 36,000× for detailed synaptic features.

### 2.8 The Enzyme‐Linked Immunosorbent Assay (ELISA)

The experimental analysis was performed using GABA and glutamate ELISA kits (Batch No. 202112) provided by Jiangsu Jingmei Biotechnology Co. Ltd. (Jiangsu, China). The specific procedures were as follows: First, rat hippocampal tissue samples were retrieved from a −80°C ultra‐low temperature freezer and homogenized thoroughly with an appropriate amount of physiological saline in an ice‐water bath. The tissue homogenate was then centrifuged at 12,000 rpm for 15 min using a high‐speed centrifuge, after which the supernatant was carefully collected. Strictly following the kit's standard operating procedures, the optical density (OD) values of glutamate and GABA in the hippocampal tissue homogenate supernatant were measured using a microplate reader. Finally, based on the established standard curve combined with sample dilution factors, the precise concentrations of glutamate and GABA in hippocampal tissues were calculated, along with their concentration ratio (Glu/GABA).

### 2.9 Western Blot (WB)

GluA1, GluA2, GluN1A, GluN2A, GluN2B, SYP, PSD‐95, ZO‐1, Occludin, Claudin‐1, and β‐actin were obtained from Ebertek Biotechnology Co. Ltd. (A11643, A11316, A7677, A0924, A3056, A6344, A7889, A0659, A5381, A21971, AC026 Wuhan, China). The hippocampal tissues from rats in each group were processed following standard Western blot protocols. After centrifugation at 12,000 rpm for 15 min at 4°C, the supernatant was collected for protein quantification using BCA assay. Standard procedures were then performed including SDS‐PAGE electrophoresis (30 μg protein loading), PVDF membrane transfer, and washing with PBS containing 0.1% Tween‐20 (PBS‐T) three times (5 min each). The membranes were blocked with 5% skim milk at room temperature for 1 h, followed by incubation with corresponding primary antibodies overnight at 4°C: GluA1 (1:1000), GluA2 (1:1000), GluN2A (1:1000), GluN2B (1:1000), SYP (1:1000), PSD‐95 (1:1000), ZO‐1 (1:1000), Occludin (1:1000), Claudin‐1 (1:500), β‐actin (1:100,000). After incubation with fluorescent secondary antibodies at room temperature (protected from light) for 2 h, a high‐resolution fluorescence imaging system was employed to capture specific fluorescence signals. The acquired images were then imported into Image J image analysis software for grayscale quantification of target bands by setting uniform thresholds and measurement parameters. All data were collected from triplicate measurements and presented as mean values.

### 2.10 Real‐Time Fluorescence Quantitative PCR (RT‐qPCR)

Following standard molecular biology protocols, rat hippocampal tissues were rapidly homogenized into fine powder using a liquid nitrogen‐prechilled mortar. Total RNA was then extracted using 1 mL TRIzol reagent via the phenol‐chloroform method. For cDNA synthesis, 1 μg of total RNA was reverse‐transcribed in a 20 μL reaction mixture under the following conditions: 37°C for 15 min (reverse transcription), 85°C for 5 s (enzyme inactivation), and 4°C for 5 min (reaction termination). A 50 μL reaction mixture was set up for RT‐PCR. The amplification protocol consisted of an initial denaturation at 95°C for 30 min, followed by 40 cycles of 95°C for 5 s and 60°C for 34 s for annealing and extension. This was succeeded by a final extension at 95°C for 15 min and 60°C for 1 h. SYBR Green was used for fluorescence‐based detection, and the relative expression levels of target genes were determined using the 2^−ΔΔCt^ method. The primer sequences are listed in Table 1.

**TABLE 1:** The primer sequences.

Primer	Sequences	Base number
GluA1‐F	GACTGTGAATCAGAACGCCTCAAC	24
GluA1‐R	GTCACATTGGCTCCGCTCTC	20
GluA2‐F	CATCCAGATGCGACCTGACC	20
GluA2‐R	CTCTGCAGCAGAATCCAGAACA	22
GluN1A‐F	ACCTCGCTCTGCTCCAGTTTG	21
GluN1A‐R	CCGCCATGTTATCGATGTCC	20
GluN2A‐F	ACCTCGCTCTGCTCCAGTTTG	21
GluN2A‐R	CCGCCATGTTATCGATGTCC	20
GluN2B‐F	GAAGCCATCGCTCAGATCCTC	21
GluN2B‐R	GATAGACGGGCCAAACTGGAA	21
SYP‐F	GACCGTTGGTAGTGCCTGTGA	21
SYP‐R	GCACAGGAAAGTAGGGGGTC	20
PSD95‐F	ACCTCGCTCTGCTCCAGTTTG	21
PSD95‐R	CCGCCATGTTATCGATGTCC	20
β‐actin‐F	GGAGATTACTGCCCTGGCTCCTA	23
β‐actin‐R	GACTCATCGTACTCCTGCTTGCTG	24

### 2.11 Hematoxylin and Eosin Staining of Colonic Tissue (H&E)

After collection, colonic tissue samples were quickly washed three times with ice‐cold phosphate‐buffered saline (PBS, pH 7.4) and then fixed in 4% paraformaldehyde (PFA) at 4°C for 24–48 h. Following gradient ethanol dehydration (70%–100%) and paraffin embedding, 5‐μm serial sections were prepared and stained using standard hematoxylin (5 min) and eosin (2 min) (H&E) protocol. After neutral balsam mounting, comprehensive histopathological evaluation was performed under light microscopy to systematically assess mucosal integrity, glandular architecture, and inflammatory cell infiltration.

### 2.12 Immunohistochemistry Staining of Colonic Tissue (IHC)

The paraffin‐embedded tissue sections underwent standard deparaffinization, followed sequentially by heat‐induced antigen retrieval, endogenous peroxidase blocking, and serum sealing. The sections were then incubated overnight at 4°C with three tight junction protein primary antibodies: Occludin (1:250), ZO‐1 (1:1000), and Claudin‐1 (1:250), followed by secondary antibody incubation and DAB chromogenic reaction. Finally, after hematoxylin nuclear counterstaining, gradient ethanol dehydration, and neutral balsam mounting, the sections were observed and imaged under an optical microscope.

### 2.13 16S rRNA Amplicon Sequencing Experimental Method (16S rRNA)

Total genomic DNA was extracted using the MagPure Soil DNA LQ Kit (Magen) following the manufacturer's protocol. For bacterial diversity analysis, the V3–V4 hypervariable regions of 16S rRNA genes were amplified using universal primers 343F (5′‐TACGGRAGGCAGCAG‐3′) and 798R (5′‐AGGGTATCTAATCCT‐3′) along with 907R (5′‐CCGTCAATTCMTTTRAGTTT‐3′). Sequencing was conducted on the Illumina NovaSeq 6000 system, producing 250 bp paired‐end reads. Library construction and subsequent data processing were performed by OE Biotech Co. Ltd. (Shanghai, China). Microbial community diversity was analyzed using QIIME2 software. Beta diversity was evaluated via principal coordinates analysis (PCoA) based on unweighted UniFrac distance matrices, utilizing R packages. Differences in taxonomic composition were examined using the linear discriminant analysis effect size (LEfSe) approach. All bioinformatics analyses were executed on the OECloud platform (https://cloud.oebiotech.com/task/), with visualizations created using R (https://www.r‐project.org/) within the OECloud environment.

### 2.14 Statistical Analysis

Statistical analyses were performed with SPSS (version 27.0). For data meeting the criteria of normality and homogeneity of variance, differences between groups were evaluated using one‐way ANOVA, followed by Tukey's post hoc test when significant differences were detected. For datasets that violated the normality assumption, the Kruskal–Wallis nonparametric test was employed. All graphs were generated using GraphPad Prism (version 10), and *p*‐values less than 0.05 were considered statistically significant.

## 3 Result

### 3.1 Network Pharmacology and Molecular Docking Results

**FIGURE 2:**
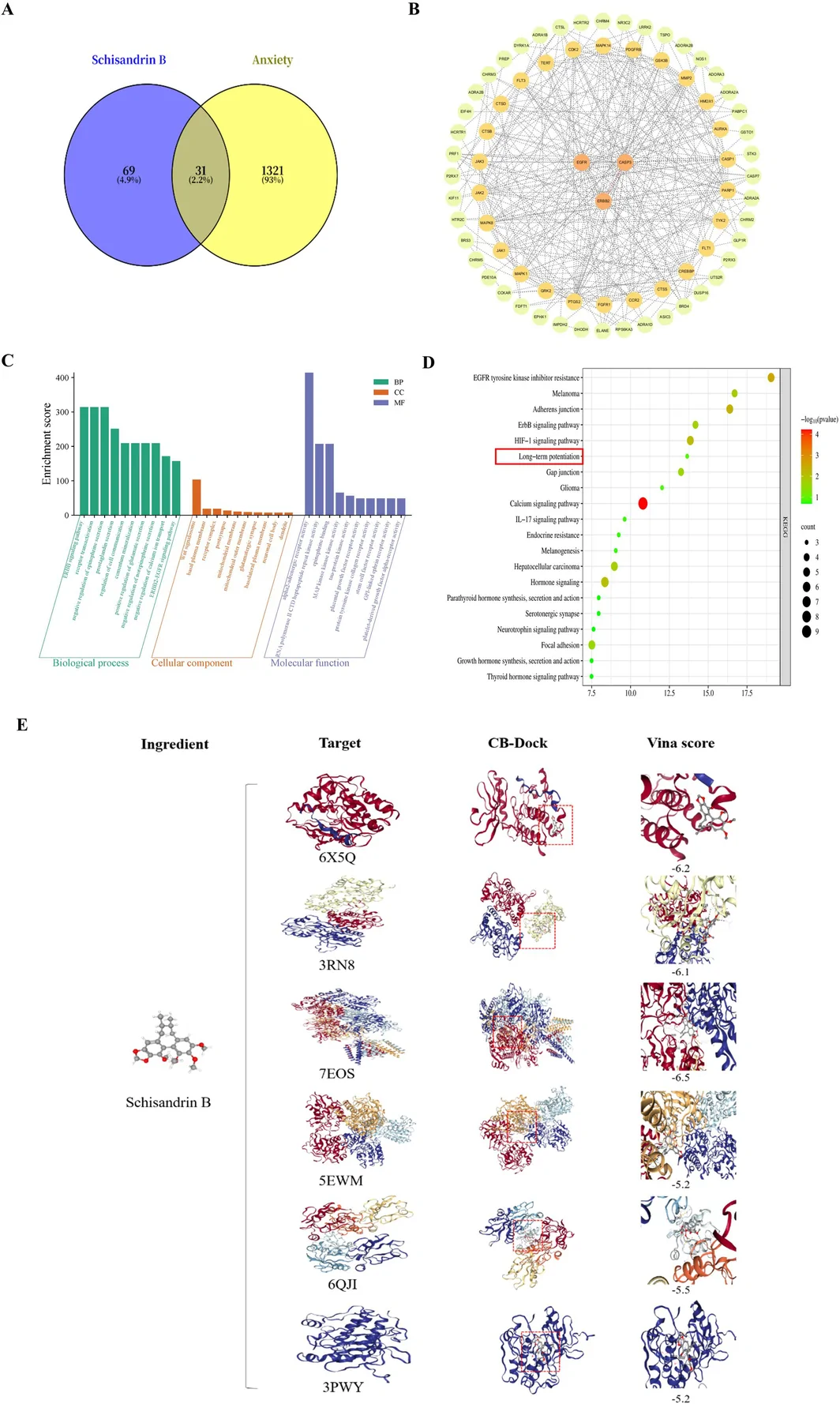
Network pharmacology analysis and molecular docking of Schisandrin B against anxiety‐related targets. (A) Venn diagram showing the intersection between putative targets of Schisandrin B and anxiety‐related targets. (B) Protein–protein interaction (PPI) network of the intersecting targets. Nodes represent target proteins, and edges indicate protein–protein interactions. Node size and color reflect topological parameters. (C) Gene Ontology (GO) enrichment analysis of the intersecting targets, including biological process (BP), cellular component (CC), and molecular function (MF). (D) KEGG pathway enrichment analysis of the intersecting targets. The size of the dots represents the number of enriched genes, and the color indicates the significance level. (E) Molecular docking results of Schisandrin B with core target proteins. Schisandrin B is shown in stick representation, and target proteins are shown in cartoon representation. The red boxes indicate the binding sites.

Putative targets of Schisandrin B were identified through multiple databases, and anxiety‐related targets were collected from the GeneCards database. Intersection analysis revealed several common targets between Schisandrin B and anxiety (Figure 2A), suggesting that Schisandrin B may exert its anti‐anxiety effects through a multi‐target regulatory mechanism. Based on these intersecting targets, a protein–protein interaction (PPI) network was constructed (Figure 2B). Topological analysis revealed that the key targets were predominantly associated with synaptic communication, glutamate‐mediated neurotransmission, inflammatory processes, and stress‐related signaling pathways, all of which are crucial in the development and progression of anxiety. In addition, Gene Ontology (GO) functional enrichment and KEGG pathway analyses were conducted on the overlapping targets (Figure 2C,D). The results suggested that Schisandrin B may alleviate anxiety by regulating synaptic structure and neurotransmitter signaling, as well as through the coordinated modulation of multiple signaling pathways involved in central nervous system function, thereby improving anxiety‐like behaviors. To further confirm the interaction between Schisandrin B and the key targets, molecular docking was performed on the targets identified through network pharmacology. The results revealed that Schisandrin B could stably fit into the active sites of these proteins and showed strong binding affinities with all core targets, suggesting its potential to regulate their activity at the molecular level (Figure 2E).

### 3.2 Anti‐Anxiety Effect of Schisandrin B on Anxiety Rats

**FIGURE 3:**
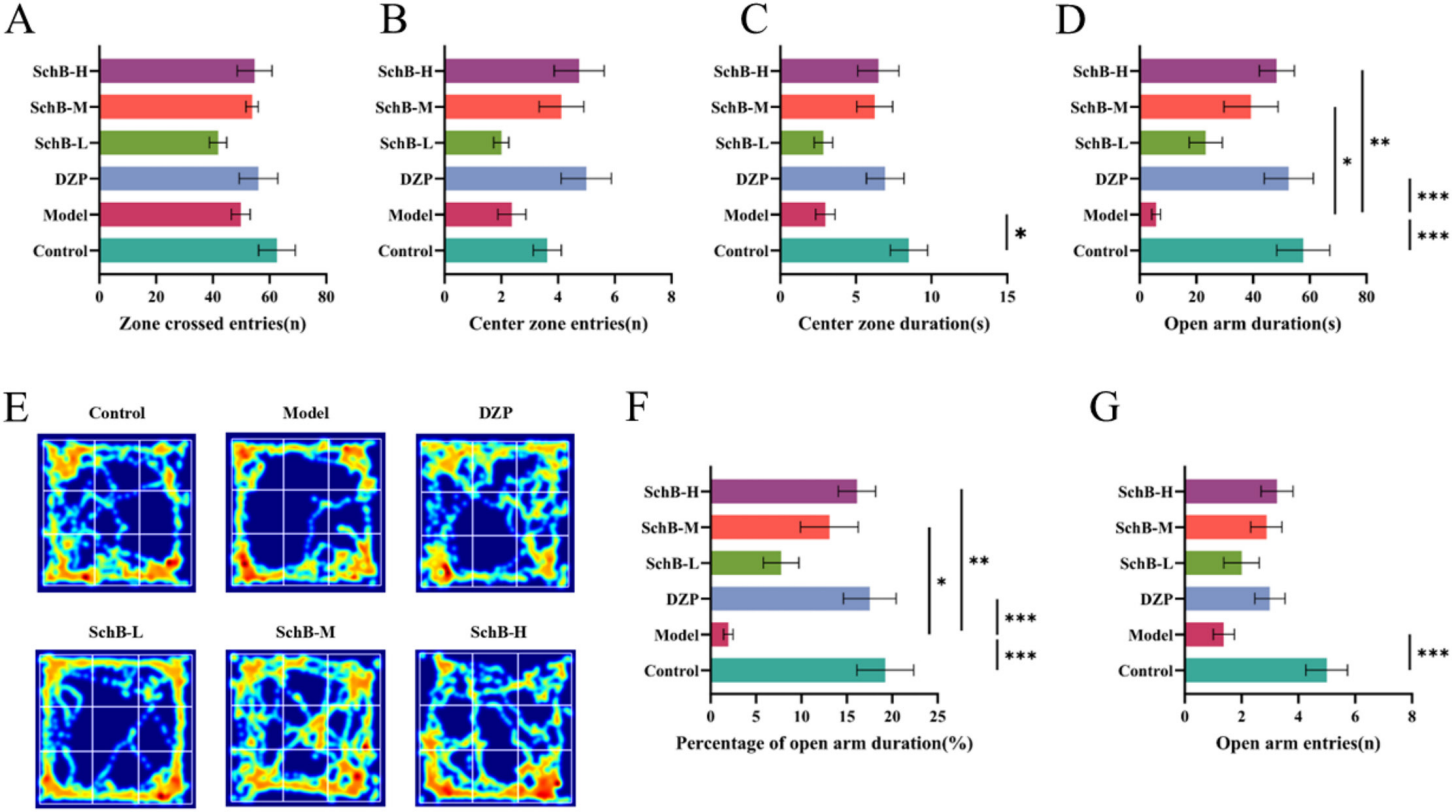
Behavioral testing results. (A) Zone crossed entries (*n*) (*n* = 8). (B) Center zone entries (*n*) (*n* = 8). (C) Center zone duration (s) (*n* = 8). (D) Open arm durations (s) (*n* = 8). (E) OFT locomotion trajectory plots. (F) Percentage of open arm duration (%) (*n* = 8). (G) Open arm entries (*n*) (*n* = 8). The data are shown as the mean ± SEM. *p < 0.05, **p < 0.01, and ***p < 0.001.

To evaluate the anxiolytic effects of SchB, behavioral assessments were performed using the open field test (OFT) and elevated plus maze (EPM) (Figure 3). In the OFT, rats in the model group exhibited fewer zone crossings and center zone entries than those in the control group, although these differences did not reach statistical significance (Figure 3A,B). In contrast, the center dwelling time was significantly reduced in the model group (Figure 3C), indicating enhanced anxiety‐like behavior. Treatment with diazepam or SchB increased these behavioral parameters to varying degrees; however, no statistically significant differences were observed compared with the model group (Figure 3A–C). In the EPM, the model group showed significantly reduced open‐arm dwelling time, percentage of open‐arm duration, and number of open‐arm entries compared with the control group (Figure 3D,F,G). Both diazepam and SchB treatment significantly improved these behavioral indices, suggesting marked anxiolytic effects (Figure 3D,F,G). Representative OFT locomotor trajectories are shown in Figure 3E. Compared with the control group, rats in the model group exhibited reduced exploration of the central area, whereas diazepam‐ and SchB‐treated rats showed increased movement within the center zone. Notably, no significant differences were observed between the SchB‐M (10 mg/kg) and SchB‐H (20 mg/kg) groups, and their behavioral outcomes were highly comparable. Therefore, the medium dose (SchB‐M, 10 mg/kg) was selected for subsequent mechanistic studies, as it provided equivalent therapeutic efficacy while minimizing potential dose‐related effects.

### 3.3 Schisandrin B Preserves Hippocampal Neurotransmitter Homeostasis and Synaptic Plasticity in Rats With Anxiety‐Like Behaviors

**FIGURE 4:**
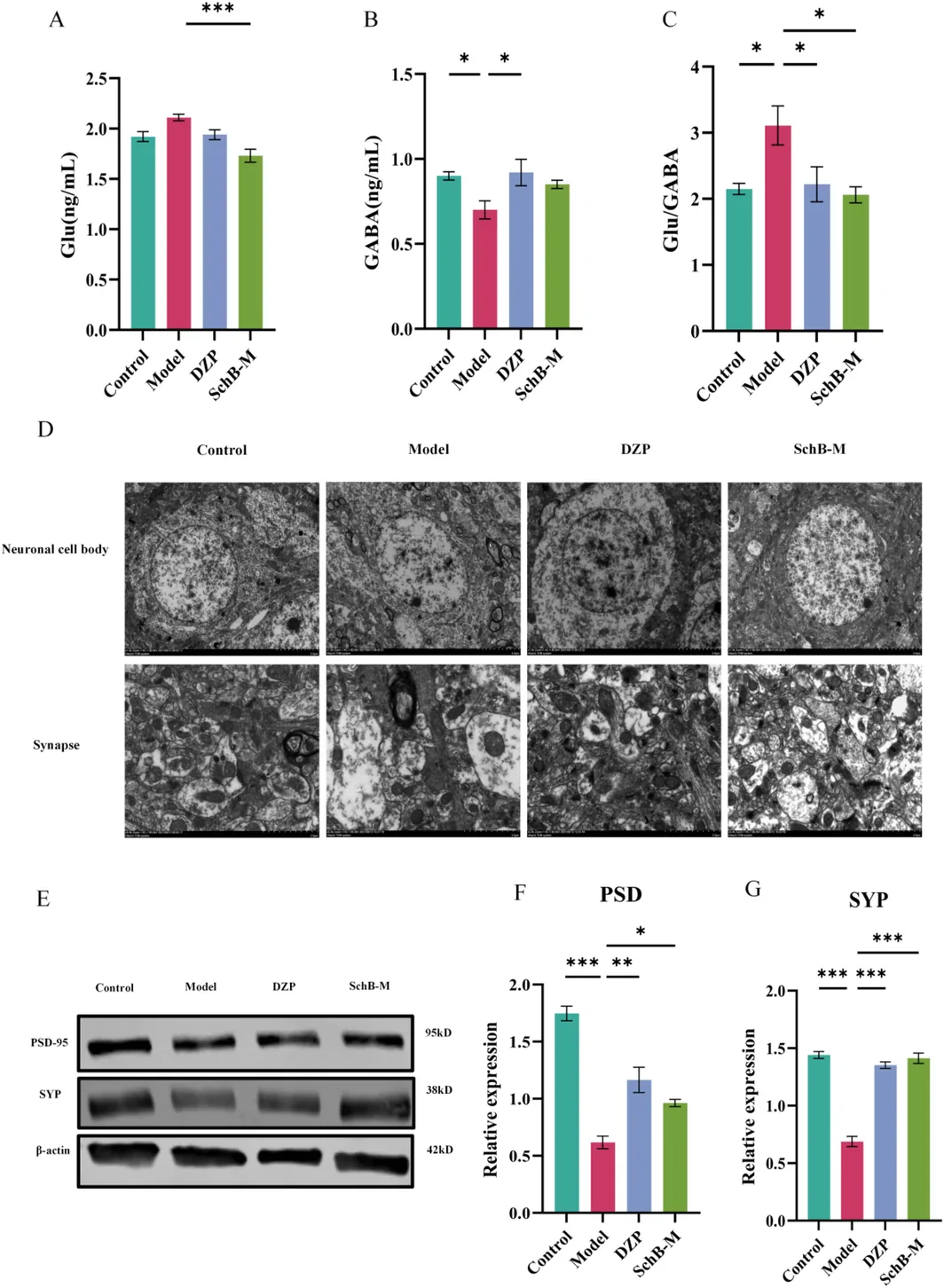
Effects of Schisandrin B on hippocampal glutamate and GABA homeostasis and synaptic plasticity in anxious rats. (A) Glutamate content in the hippocampus (ng/mL). (B) GABA content in the hippocampus (ng/mL). (C) Glutamate/GABA ratio in the hippocampus. (D) The ultrastructural features of synapses in the hippocampal region of each group of rats (Scale bar = 2 μm, ×36,000), and the ultrastructural features of synapses in the hippocampal region of each group of rats (Scale bar = 2 μm, ×36,000). (E) Western blot analysis of PAD‐95 and SYP expression in the hippocampal region. The data are shown as the mean ± SEM. *p < 0.05, **p < 0.01, and ***p < 0.001.

Compared to the control group, the model group displayed an increase in hippocampal Glutamate levels, though the difference was not statistically significant (Figure 4A). In contrast, GABA levels were significantly lower in the model group (Figure 4B). Treatment with SchB led to a significant reduction in Glutamate levels compared with the model group (Figure 4A), while GABA levels exhibited a slight, non‐significant upward trend (Figure 4B). The Glu/GABA ratio was significantly increased in the model group compared to controls, with both diazepam and SchB treatments significantly reducing this ratio (Figure 4C). These findings imply that Schisandrin B may confer neuroprotective effects via modulation of the GABAergic and glutamatergic systems, although the underlying mechanisms warrant further investigation.

Transmission electron microscopy revealed marked ultrastructural alterations in the hippocampus of anxiety model rats (Figure 4D). Compared with the control group, which exhibited intact neuronal morphology, clear nuclear and plasma membranes, abundant organelles, and well‐preserved synaptic structures, the model group showed obvious neuronal swelling, nuclear membrane disruption, cytoplasmic vacuolization, organelle loss, and severe synaptic damage. Synaptic abnormalities included blurred synaptic contours, indistinct pre‐ and postsynaptic membranes, and a marked reduction in synaptic vesicle density. In contrast, both diazepam and SchB treatment markedly improved neuronal and synaptic morphology. Neurons displayed clearer cellular structures with restored organelle distribution, while synapses exhibited well‐defined synaptic clefts and increased synaptic vesicle density. The ultrastructural improvements observed in the SchB‐treated group were comparable to those in the control group.

To further evaluate synaptic plasticity, the protein expression levels of SYP and PSD‐95 in hippocampal tissues were determined by Western blotting. Compared with the control group, the model group showed significantly reduced expression of both SYP and PSD‐95 (Figure 4E–G). Treatment with diazepam or SchB significantly reversed these decreases. These findings suggest that SchB may protect hippocampal neuronal integrity and synaptic plasticity by maintaining the expression of key synaptic proteins.

### 3.4 Effects of Schisandrin B on Hippocampal NMDAR and AMPAR Expression and HPA Axis Activity in Anxiety Rats

To investigate the effects of Schisandrin B on ionotropic glutamate receptors in the hippocampus, the protein and mRNA expression levels of NMDAR subunits (GluN1A, GluN2A, and GluN2B) and AMPAR subunits (GluA1 and GluA2) were assessed by Western blotting and qRT‐PCR.

**FIGURE 5:**
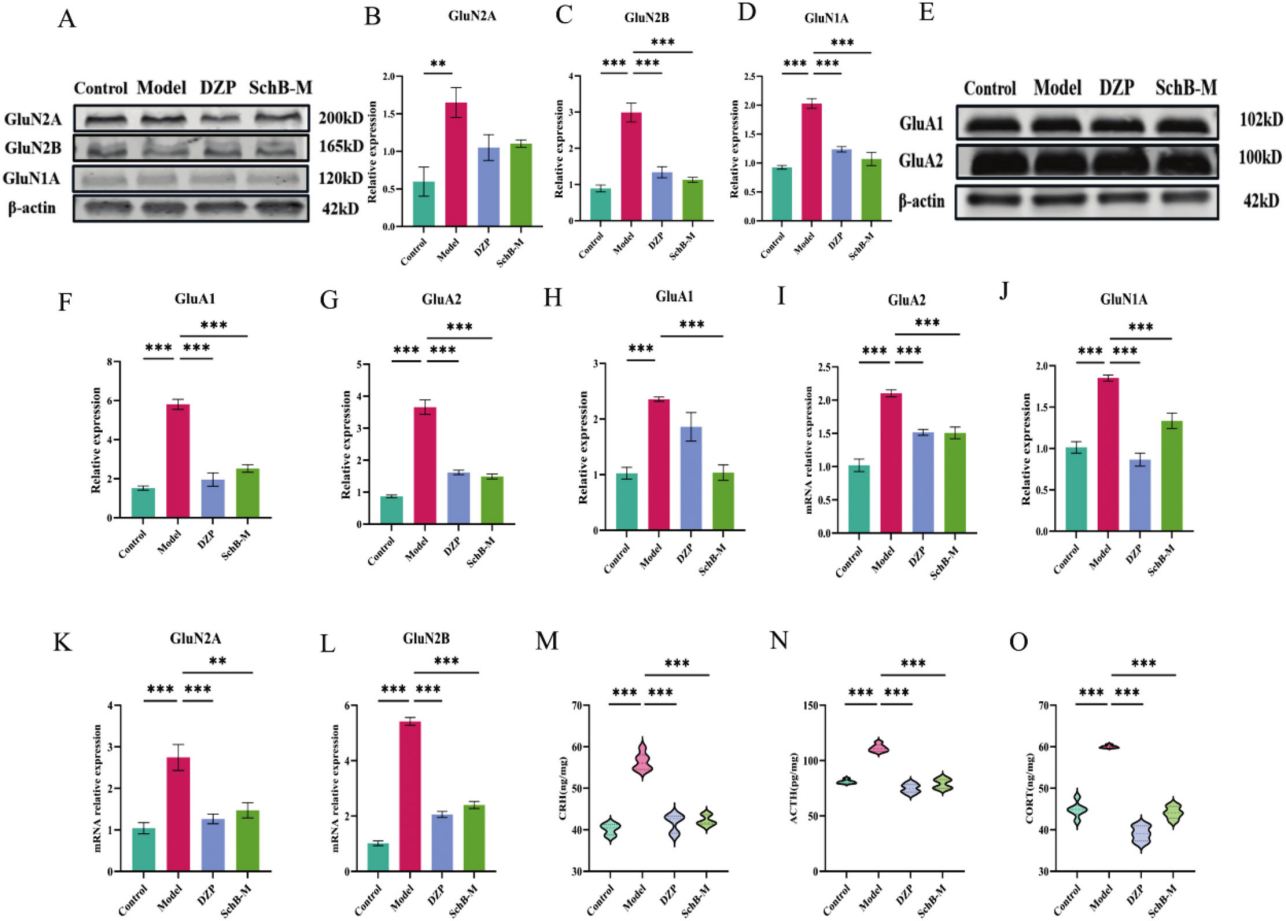
Expression of NMDAR and AMPAR subunit proteins and mRNAs in the hippocampus, and serum levels of HPA axis hormones (CRH, ACTH, and CORT). (A) Western blot analysis of NMDAR subunit expression in the hippocampal region. (B) Relative expression of GluN2A protein across experimental groups. (C) Relative expression of GluN2B protein across experimental groups. (D) Relative expression of GluN1A protein across experimental groups. (E) Western blot analysis of AMPAR subunit expression in the hippocampal region. (F) Relative expression of GluA1 protein across experimental groups. (G) Relative expression of GluA2 protein across experimental groups. (H) Relative expression levels of GluA1 mRNA in the hippocampal region. (I) Relative expression levels of GluA2 mRNA in the hippocampal region. (J) Relative expression levels of GluN1A mRNA in the hippocampal region. (K) Relative expression levels of GluN2A mRNA in the hippocampal region. (L) Relative expression levels of GluN2B mRNA in the hippocampal region. (M) CRH concentration in serum. (N) ACTH concentration in serum. (O) CORT concentration in serum. The data are shown as the mean ± SEM. **p < 0.01, and ***p < 0.001.

Compared with the control group, the anxiety model group exhibited significantly elevated protein and mRNA expression levels of GluN1A, GluN2A, and GluN2B in the hippocampus (Figure 5A–D,J–L). Treatment with diazepam significantly reduced the expression of these NMDAR subunits. Similarly, SchB administration markedly decreased the expression levels of GluN1A, GluN2A, and GluN2B compared with the model group (Figure 5A–D,J–L).

A similar pattern was observed for AMPAR subunits. The protein and mRNA expression levels of GluA1 and GluA2 were significantly higher in the model group than in the control group (Figure 5E–I). Both diazepam and SchB treatment significantly attenuated the upregulation of GluA1 and GluA2 induced by anxiety (Figure 5E–I).

These findings indicate that Schisandrin B effectively reversed the anxiety‐associated upregulation of NMDAR and AMPAR subunits in the hippocampus.

The model group showed markedly higher levels of CRH, ACTH, and CORT compared to the blank control group (Figure 5M–O). In contrast, both the DZP and SchB‐M groups showed significantly reduced levels of these HPA axis hormones compared to the model group (Figure 5M–O). These findings demonstrate that Schisandrin B can effectively regulate HPA axis‐related hormone levels, confirming its modulatory effects on neuroendocrine stress responses.

### 3.5 Effects of Schisandrin B on Colon Morphology and Colonic Tight Junction Protein Expression in Anxious Rats

**FIGURE 6:**
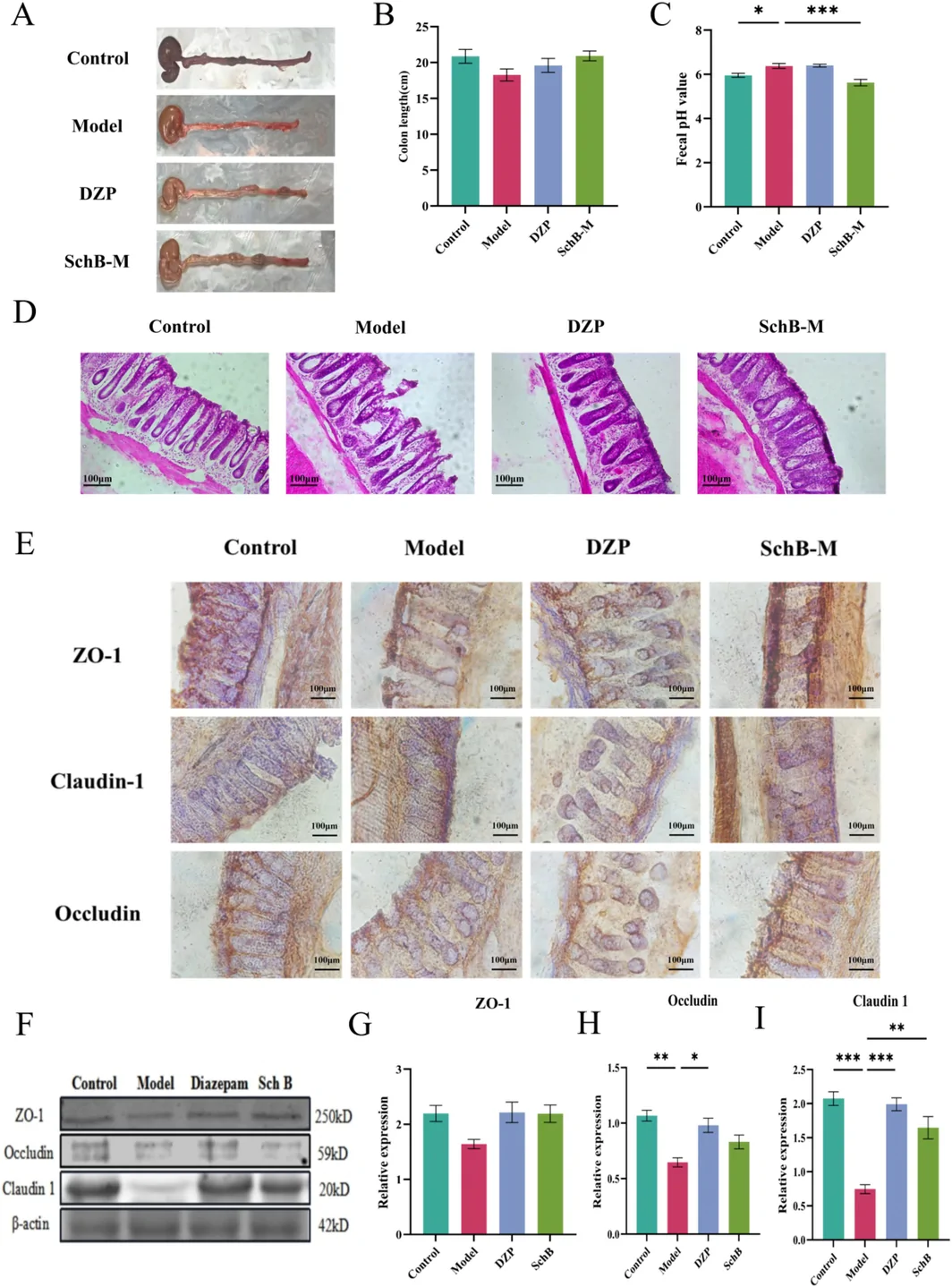
Colonic tissue structural changes and detection of tight junction proteins. (A) Colon length photograph. (B) Colon length. (C) Fecal pH value. (D) Histopathological analysis of colonic tissue by H&E staining (scale bar = 100 μm, 200×). (E) IHC staining results of tight junction proteins (ZO‐1, Claudin‐1, Occludin) in colonic tissue (scale bar = 100 μm, 200×). (F) Western blot analysis of tight junction proteins (ZO‐1, Claudin‐1, Occludin) in colonic tissue. (G) Relative expression of ZO‐1 protein across experimental groups. (H) Relative expression of occludin protein across experimental groups. (I) Relative expression of Claudin‐1 protein across experimental groups. The data are shown as the mean ± SEM. *p < 0.05, **p < 0.01, and ***p < 0.001.

Histopathological examination of rat colons revealed that compared with the blank control group, the model group exhibited colonic shortening (Figure 6A,B). Although both the DZP and SchB‐M groups showed some degree of length recovery relative to the model group, these improvements were not statistically significant (Figure 6A,B). The model group demonstrated significantly elevated fecal pH compared to controls, which was significantly reduced following SchB treatment. HE staining results (Figure 6D) showed that the blank control group maintained intact intestinal villi structure with well‐organized arrangement. In contrast, the model group displayed marked intestinal barrier damage characterized by partial villus loss, disrupted crypt architecture, significantly reduced goblet cells, and evident inflammatory infiltration. Notably, both the DZP and SchB‐M groups exhibited varying degrees of mucosal layer restoration with improved histological architecture compared to the model group.

Immunohistochemical analysis revealed that in the control group, ZO‐1, Claudin‐1, and Occludin were predominantly localized at the tight junctions of intestinal villi. Compared with controls, the conditional fear‐induced anxiety model group showed markedly weakened expression intensity and dispersed distribution of these tight junction proteins in colonic tissues, while the SchB group exhibited enhanced expression of these proteins (Figure 6E). Western blot analysis revealed that, compared with the control group, the model group showed a downward trend in ZO‐1 protein expression, although this change was not statistically significant (Figure 6G). In contrast, both Occludin and Claudin‐1 levels were significantly decreased (Figure 6H,I). The DZP treatment resulted in a non‐significant increase in ZO‐1 expression but caused significant upregulation of Occludin and Claudin‐1 compared to the model group (Figure 6F–I). Similarly, the SchB‐M group exhibited a significant rise in Claudin‐1 levels and showed upward trends in ZO‐1 and Occludin expression, although these were not statistically significant (Figure 6F–I).

### 3.6 16S rRNA

**FIGURE 7:**
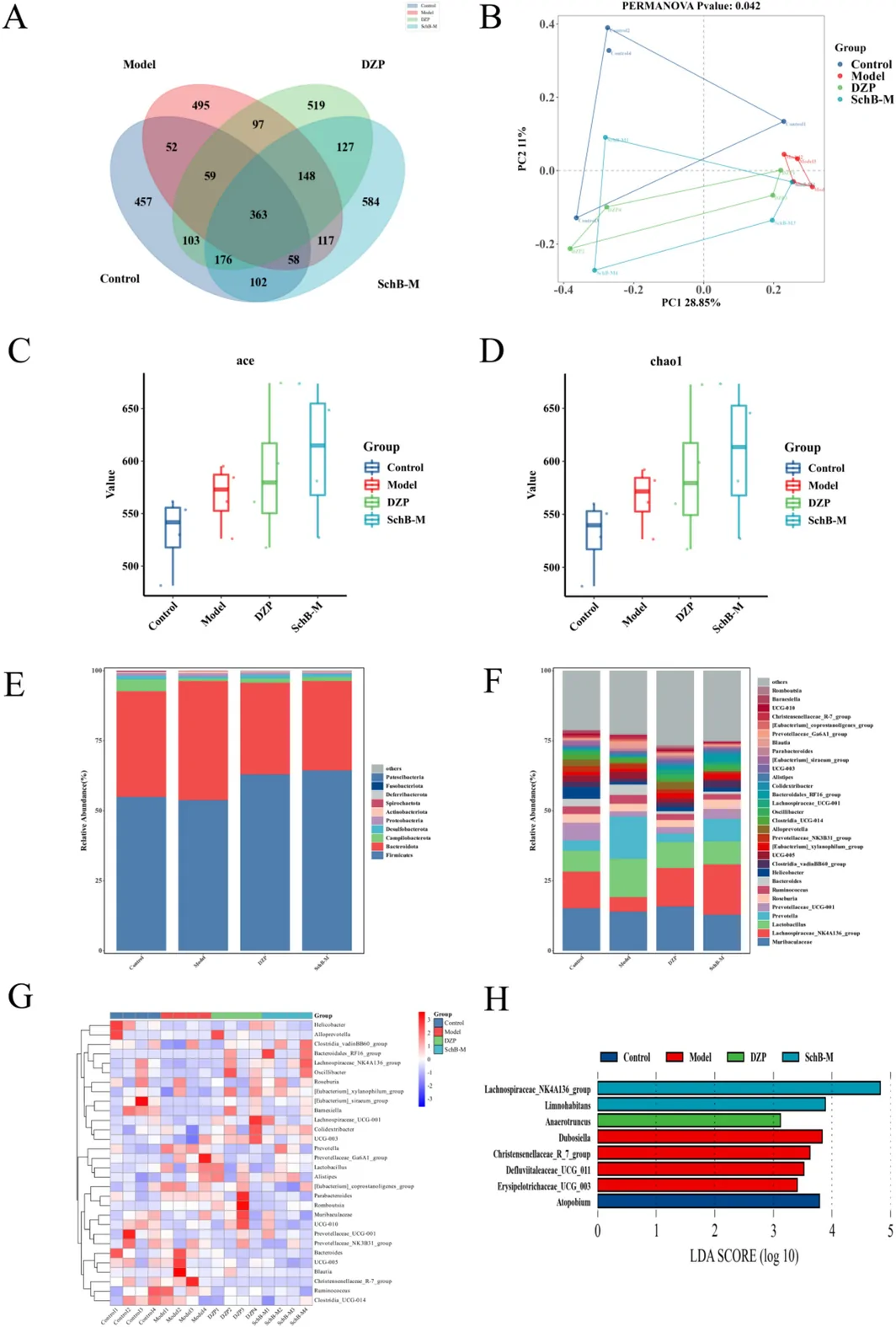
Schisandrin B modulates the gut microbiota structure in rats with conditioned fear‐induced anxiety. (A) Venn diagram showing distribution of amplicon sequence variants. (B) Principal coordinates analysis based on Bray‐Curtis distances. (C) Comparison of ACE indices for microbial alpha diversity. (D) Chao1 index of microbial community richness. (E) Phylum‐level community composition bar plot. (F) Genus‐level community composition bar plot. (G) Heatmap showing genus‐level composition of gut microbiota. (H) LEfSe analysis plot showing differentially abundant taxa across groups. The data are shown as the mean ± SEM. *p < 0.05, **p < 0.01, and ***p < 0.001.

16S rRNA gene sequencing analysis revealed that both the conditioned fear model and Schisandrin B intervention significantly altered the overall composition of gut microbiota. Specifically, only 363 amplicon sequence variants (ASVs) were shared among all four mouse groups, with the Control group containing 457 unique ASVs. The Model, DZP, and SchB‐M groups showed values of 459, 519, and 584, respectively (Figure 7A). Notable differences in gut microbiota diversity were observed among groups: Principal coordinate analysis (PCoA) of beta diversity demonstrated distinct clustering of microbial communities induced by conditioned fear‐related anxiety and SchB intervention (Figure 7B). Alpha diversity analysis indicated that the Model group exhibited distinct microbial richness and diversity compared to other groups, while SchB showed partial ameliorative effects on dysbiosis (with ACE and Chao1 indices reflecting species richness diversity) (Figure 7C,D).

At the phylum level, the gut microbiota was primarily composed of Firmicutes and Bacteroidetes, with the Model group showing a greater proportion of Bacteroidetes compared to the other groups (Figure 7E). At the genus level, Prevotella and Lactobacillus were significantly more abundant in the Model group than in the remaining three groups (Figure 7F). Heatmap analysis at the genus level (Figure 7G) showed that conditioned fear‐induced anxiety primarily increased the abundances of *Prevotella*, *Prevotella‐Ga6A1‐group*, *Lactobacillus*, and *Parabacteroides*, while decreasing the relative abundances of *Prevotellaceae_UCG‐001* and *Barnesiella* compared to the Control group. Furthermore, the SchB group exhibited increased relative abundances of *Alistipes* and *Lachnospiraceae‐NK4A136‐group*, along with decreased abundances of *Parabacteroides*, *Lactobacillus*, *Christensenellaceae‐R‐7‐group*, *Ruminococcus*, and *Clostridia‐UCG‐014* relative to the Model group (Figure 7G). LEfSe analysis identified *Lachnospiraceae‐NK4A136‐group* as the predominant bacterial genus following SchB treatment (Figure 7H).

## 4 Discussion

In the present study, Schisandrin B was administered at three doses (5, 10, and 20 mg/kg) to evaluate its anxiolytic efficacy. Behavioral assessments showed that both the medium‐ and high‐dose groups significantly improved anxiety‐like behaviors, whereas no significant differences were observed between the two groups. Therefore, the 10 mg/kg dose was selected for subsequent mechanistic studies because it achieved comparable efficacy while reducing compound consumption and potential dose‐related risks. Nevertheless, the current experimental design does not allow determination of the minimum effective dose or the precise dose–response relationship of Schisandrin B. Future studies employing a broader range of doses and pharmacokinetic analyses are warranted to identify the optimal therapeutic window.

Safety evaluation is an important consideration for the future development of Schisandrin B as a therapeutic candidate. Previous studies have reported that Schisandrin B exhibits favorable safety profiles and antioxidant activities in experimental models, with no obvious toxic effects observed at pharmacologically relevant doses (Feng et al. 2018). However, the present study focused primarily on the anxiolytic efficacy and underlying mechanisms of Schisandrin B and did not include comprehensive toxicological assessments, such as serum biochemical analyses or histopathological examinations of major organs. Therefore, further investigations are required to systematically evaluate its potential hepatotoxicity, nephrotoxicity, and long‐term safety before clinical translation.

Anxiety disorders are strongly linked to central nervous system abnormalities, with the hippocampus playing a key role in managing stress and processing emotions, serving as an important hub in neural circuits associated with anxiety. (Ellard et al. 2017). Transmission electron microscopy revealed pronounced ultrastructural damage of hippocampal synapses in anxious rats, whereas Schisandrin B treatment markedly restored synaptic integrity and structural clarity, suggesting a protective effect on synaptic structural plasticity. Synaptic plasticity is fundamental to neuronal functional regulation, and its structural and functional impairments are considered important pathological bases of various neuropsychiatric disorders (Kandola et al. 2019; Kavalali and Monteggia 2020). Further molecular evidence showed that Schisandrin B significantly reversed the anxiety‐induced downregulation of the presynaptic protein synaptophysin (SYP) and the postsynaptic density protein PSD‐95, indicating that it may enhance hippocampal neural network stability by improving synaptic connectivity and information transmission.

At the neurotransmitter level, anxiety model rats exhibited a typical imbalance between excitatory and inhibitory transmission, characterized by elevated glutamate levels, reduced GABA levels, and an increased Glu/GABA ratio. Schisandrin B treatment markedly reversed these abnormalities, thereby maintaining the dynamic balance between excitatory and inhibitory neurotransmission in the hippocampus. As major mediators of central excitatory synaptic transmission, ionotropic glutamate receptors, including NMDARs and AMPARs, play critical roles in the regulation of anxiety‐related behaviors (Sedlácek et al. 2008). In the present study, both mRNA and protein expression levels of hippocampal NMDAR subunits (GluN2A and GluN2B) and AMPAR subunits (GluA1 and GluA2) were significantly upregulated under anxiety conditions, whereas Schisandrin B effectively suppressed this excessive receptor expression. Previous studies have demonstrated that overactivation of NMDA receptors exacerbates anxiety‐like behaviors, while NMDA receptor antagonists such as MK‐801 exhibit clear anxiolytic effects (Kocahan and Akillioglu 2013; Salunke et al. 2014). Therefore, these findings suggest that Schisandrin B may exert anxiolytic effects by attenuating glutamate receptor–mediated excessive excitatory transmission and improving hippocampal neural circuit function.

In addition to its role in emotional regulation, the hippocampus is also a crucial center for negative feedback control of the hypothalamic–pituitary–adrenal (HPA) axis (Leuner and Gould 2010). Impairment of hippocampal function disrupts HPA axis negative feedback, leading to sustained glucocorticoid elevation and exacerbated stress responses. In this study, anxiety model rats exhibited significantly elevated levels of HPA axis–related hormones, whereas Schisandrin B treatment restored these levels toward those of the normal control group, consistent with previous findings (Li et al. 2025). These results further indicate that Schisandrin B may alleviate chronic stress–induced anxiety responses by improving hippocampal functional status and suppressing HPA axis hyperactivation.

Notably, the beneficial effects of Schisandrin B on hippocampal function, HPA axis activity, and intestinal barrier integrity may represent interconnected processes rather than independent events. The hippocampus serves as an important regulator of HPA axis negative feedback. Under chronic stress conditions, excessive glutamatergic signaling and synaptic dysfunction in the hippocampus may impair this regulatory capacity, leading to persistent activation of the HPA axis and elevated secretion of stress hormones. Sustained glucocorticoid exposure can subsequently disrupt intestinal tight junctions, increase intestinal permeability, and promote peripheral inflammatory responses. These peripheral alterations may further affect central neurotransmission through the microbiota–gut–brain axis, thereby aggravating anxiety‐related behaviors. Therefore, the present findings suggest that Schisandrin B may exert anxiolytic effects through a coordinated regulatory network linking hippocampal synaptic plasticity, neuroendocrine homeostasis, and intestinal barrier function.

Emerging evidence indicates that emotional disorders and chronic stress not only affect central nervous system function but also disturb peripheral physiological homeostasis, particularly intestinal barrier function (de Lima et al. 2025). Under stress conditions, excessive activation of the HPA axis leads to persistently elevated glucocorticoid levels, which downregulate tight junction protein expression, increase intestinal permeability, and promote inflammatory factor release (Chen et al. 2021; Liu et al. 2020). Impairment of intestinal barrier integrity is therefore considered an important peripheral manifestation of anxiety‐ and stress‐related pathological changes.

In the present study, the expression levels of intestinal tight junction proteins, including Occludin, Claudin‐1, and ZO‐1, were significantly reduced in anxiety model rats, indicating disruption of intestinal barrier integrity. Schisandrin B intervention markedly restored the expression of these proteins, suggesting that it effectively improves intestinal epithelial barrier function and reduces intestinal permeability. These findings indicate that, in addition to alleviating anxiety‐related pathological states, Schisandrin B exerts protective effects against stress‐induced intestinal barrier damage.

Furthermore, gut microbiota analysis showed that Schisandrin B significantly modulated the relative abundance of Bacteroidetes‐ and Firmicutes‐related taxa, which are considered crucial for maintaining intestinal microecological homeostasis and regulating inflammation and metabolic balance (Nikel et al. 2025). Previous studies have reported that certain gut microbes can influence host stress responses and inflammatory status through the production of metabolites such as GABA and short‐chain fatty acids (Cao et al. 2025; Nikel et al. 2025). Therefore, the present findings suggest that regulation of gut microbiota composition, together with improvement of intestinal barrier integrity, may contribute to the mitigation of stress‐related peripheral inflammation and endocrine disturbances, thereby supporting the overall anxiolytic effects of Schisandrin B.

Although the anxiolytic efficacy of Schisandrin B was generally comparable to that of diazepam in the present study, its potential value may extend beyond symptom control alone. Diazepam is a widely used first‐line anxiolytic agent that produces rapid therapeutic effects primarily through potentiation of GABAergic neurotransmission. However, long‐term use of benzodiazepines is often associated with adverse effects, including sedation, tolerance, dependence, and withdrawal symptoms. In contrast, the present findings suggest that Schisandrin B exerts broader regulatory effects involving hippocampal synaptic plasticity, glutamatergic signaling, HPA axis activity, intestinal barrier integrity, and gut microbiota homeostasis. Such multi‐target modulation may provide advantages in restoring systemic homeostasis and addressing multiple pathological processes associated with chronic anxiety. Therefore, Schisandrin B may represent a promising complementary or alternative therapeutic strategy deserving further investigation.

In summary, this study systematically elucidates the anxiolytic effects of Schisandrin B at multiple levels, including behavioral performance, central synaptic plasticity, excitatory/inhibitory neurotransmitter balance, HPA axis functional status, and intestinal barrier integrity. Taken together, the findings support a mechanistic framework in which Schisandrin B improves hippocampal synaptic function and glutamatergic homeostasis, thereby suppressing HPA axis hyperactivity and alleviating stress‐induced intestinal barrier dysfunction. Through this integrated regulation of the microbiota–gut–brain axis, Schisandrin B may ultimately contribute to the attenuation of anxiety‐like behaviors.

## 5 Limitation

Several limitations of the present study should be acknowledged. First, only male rats were included in the experimental design. Given the well‐documented sex differences in the prevalence, neuroendocrine regulation, and pathophysiological mechanisms of anxiety disorders, our findings may not fully represent responses in female subjects. Thus, caution is necessary when extrapolating these results across sexes. Future studies incorporating female animal models are needed to determine whether Schisandrin B exerts sex‐dependent anxiolytic effects and to improve the translational relevance of our findings. Second, although three doses of Schisandrin B were evaluated in behavioral experiments, the minimum effective dose and the complete dose–response relationship remain to be established. Moreover, comprehensive toxicological assessments were not performed and warrant further investigation. Third, the open field test apparatus used in this study did not provide total distance or average speed measurements. Therefore, we cannot completely exclude the possibility that the observed anxiolytic effects were influenced by changes in general locomotor activity, although the elevated plus maze parameters (e.g., open‐arm time) are relatively less affected by motor function. Finally, this study did not quantify colonic inflammatory cytokines or myeloperoxidase activity, which could further elucidate the link between gut microbiota changes, intestinal barrier function, and anxiety‐like behaviors. These aspects should be addressed in future research.

## 6 Conclusion

The findings of this study demonstrate the potential therapeutic effects of Schisandrin B in ameliorating anxiety‐like behaviors in rats. These beneficial effects may be attributed to multiple mechanisms: maintaining the balance of the glutamatergic system and HPA‐axis neurotransmitters in the hippocampus of anxious rats, including downregulation of ionotropic glutamate receptors (NMDAR and AMPAR); alleviating synaptic and neuronal damage while modulating the expression of synaptic plasticity markers (SYP and PSD‐95); reshaping gut microbiota composition and abundance, thereby improving intestinal barrier dysfunction and subsequently mitigating anxiety‐like behaviors through the MGB axis. Schisandrin B alleviates anxiety by simultaneously regulating multiple central neural pathways and maintaining intestinal barrier function, highlighting its promise as a multi‐target treatment for anxiety disorders.

## Author Contributions

**Jia‐Yu Tian:** writing – original draft, conceptualization, methodology, formal analysis, visualization, data curation. **Rui‐Ze Wang:** conceptualization, methodology, investigation, formal analysis. **Hong‐Kun Wang:** conceptualization, methodology, investigation, formal analysis. **Xiao‐Nan Ye:** writing – review and editing, formal analysis, investigation. **Ruo‐Xuan Li:** writing – review and editing, formal analysis. **Zi‐Hao Wang:** investigation, formal analysis. **Li‐Li Huang:** project administration, supervision. **Yan‐Yan Wang:** writing – review and editing, project administration, supervision, methodology, funding acquisition, resources.

## Funding

This research was funded by the National Natural Science Foundation of China (No. 82003974); the Natural Science Foundation of Heilongjiang Province (LH2022H086); Project of Heilongjiang Administration of Chinese Medicine (ZHY2023‐003). Heilongjiang University of Chinese Medicine Graduate Innovative Research Project (2026yjscx018).

## Conflicts of Interest

The authors declare no conflicts of interest.

## Data Availability

The data that support the findings of this study are available from the corresponding author upon reasonable request.
